# Deep Sequencing of Target Linkage Assay-Identified Regions in Familial Breast Cancer: Methods, Analysis Pipeline and Troubleshooting

**DOI:** 10.1371/journal.pone.0009976

**Published:** 2010-04-02

**Authors:** Juan Manuel Rosa-Rosa, Francisco Javier Gracia-Aznárez, Emily Hodges, Guillermo Pita, Michelle Rooks, Zhenyu Xuan, Arindam Bhattacharjee, Leonardo Brizuela, José M. Silva, Gregory J. Hannon, Javier Benitez

**Affiliations:** 1 Human Genetics Group, Spanish National Cancer Research Centre (CNIO), Madrid, Spain; 2 Howard Hughes Medical Institute, Cold Spring Harbor Laboratory (CSHL), Cold Spring Harbor, New York, United States of America; 3 Genotyping Unit (CEGEN), Spanish National Cancer Research Centre, Madrid, Spain; 4 Department of Molecular and Cell Biology, The University of Texas, Richardson, Texas, United States of America; 5 Agilent Technologies, Inc., Santa Clara, California, United States of America; 6 Irving Cancer Research Center, New York, New York, United States of America; Deutsches Krebsforschungszentrum, Germany

## Abstract

**Background:**

The classical candidate-gene approach has failed to identify novel breast cancer susceptibility genes. Nowadays, massive parallel sequencing technology allows the development of studies unaffordable a few years ago. However, analysis protocols are not yet sufficiently developed to extract all information from the huge amount of data obtained.

**Methodology/Principal Findings:**

In this study, we performed high throughput sequencing in two regions located on chromosomes 3 and 6, recently identified by linkage studies by our group as candidate regions for harbouring breast cancer susceptibility genes. In order to enrich for the coding regions of all described genes located in both candidate regions, a hybrid-selection method on tiling microarrays was performed.

**Conclusions/Significance:**

We developed an analysis pipeline based on SOAP aligner to identify candidate variants with a high real positive confirmation rate (0.89), with which we identified eight variants considered candidates for functional studies. The results suggest that the present strategy might be a valid second step for identifying high penetrance genes.

## Introduction

Breast cancer (BC [MIM #114480]) is the most frequent malignancy among women, with approximately one million new cases per year around the world [1]. About five percent of all BC cases are considered to be hereditary, and mutations in either the *BRCA1* [MIM +113705] or the *BRCA2* [MIM +600185] gene account for 25–30% of these cases [2]. Thus, about 70% of BC families remain unexplained, and are known as non-BRCA1/2 families [3]. In this regard, several linkage studies have been performed during the last years on familial BC, and many candidate regions that may contain BC susceptibility genes have been described. However, mutational screenings in linkage assay-identified regions using the classical candidate-gene approach did not identify any clear pathogenic variants [4], [5], [6]. Therefore, new strategies seem to be necessary.

Massive parallel sequencing technology allows nowadays the development of studies unachievable a few years ago. Despite the fact that the advantages of this technology were evidenced in each of the published studies based on it, the analysis of all data generated by this process remains a hard task to face. The first step in the regular analysis protocol is based on the alignment of millions of short sequences obtained from each run. For that reason, during the past years many computer tools have been developed to improve the accuracy of this process [7], [8], [9]. One of the main obstacles is the specificity in the analysis of the data to obtain the output required by any given study. For example, the identification of novel variants, chromosomal translocations, or transcription factor target sites are some of the aims of the many studies that can be performed using high throughput sequencing technologies, and each of them would require a specific analysis pipeline.

A major success in the use of this technology was the re-sequencing of the whole human genome, published in several studies in the past years [10], [11], [12], [13], [14]. The results showed a higher complexity level of the human genome, with the appearance of many new variants, short length insertions and deletions and, even, small inversions. However, the amount of time required for re-sequencing large genomes and its high economic cost make it impractical as a common laboratory technique. For that reason, the search for specific sequence-enrichment protocols applicable to massive parallel sequencing has been an important goal during these years. One of the approaches developed [15] and improved [16] to obtain this specific enrichment is the technique of selective exon-capture or hybrid selection, based on high-density tiling DNA microarrays.

In a previous study, we performed a SNP-based linkage analysis in 41 non-BRCA1/2 families and obtained several candidate regions for containing BC susceptibility genes [17]. The large size of the candidate regions and the high number of genes described within them, led us to couple linkage analysis with high-throughput sequencing-based mutational screening as a new strategy for variation detection. In the present study, we used hybrid selection of discrete genomic intervals on custom-designed microarrays as an exon-specific enrichment of all the genes located within two complete candidate regions on chromosomes 3 and 6, that presented a suggestive linkage LOD score (LOD>2.2). We also describe the complex analysis pipeline based on SOAP aligner, developed to analyse all the data obtained, and present eight variants that are candidates for playing a role in the development of familial BC.

## Results

### Regions and capture

We performed a complete mutational screening via single-end massive parallel sequencing technology on the 128 known genes located on two different chromosomal regions, previously described as candidates for containing a breast cancer susceptibility gene [17]. The first region is located on 3q25, extends over 10.8 Mb (from 160,964 to 171,786 Kb), and contains 69 known genes; the second region is located on 6q24 and spans 6.5 Mb (from 146,078 to 152,515 Kb), containing 59 known genes. To selectively sequence the coding region of these genes, we used hybrid selection on tiling microarrays [16], which was validated via qPCR (data not shown).

### Reads, coverage and depth

DNA samples from 20 affected individuals, belonging to 9 different non-BRCA1/2 families, and 4 healthy unrelated individuals from the control population were used for the analysis process. More than 102 million reads were obtained from the affected individuals and almost 22.4 million reads were obtained from the control individuals (see Table 1). The average number of reads per affected individual was 5.14 million, and this number was increased to 5.21 million when control individuals were taken into account. We used SOAP v1.0 to align our data set, obtaining an average of 91.58% aligned sequences against the whole genome with ≤2 mismatches. Among these, an average of 39.99% matched our candidate coding regions. Thus, since the total number of base pairs covered by the tiling array is 0.014% of the genome length (434,039 bp), the average enrichment was approximately 2.85 thousand times, calculated as the percentage of the sequences aligned to the genome that successfully matched to the candidate regions (39.99) divided by the percentage of the genome length that candidate regions represent (0.014).

**Table 1:** Summary of high throughput sequencing data.

			Number of sequences			Depth		
Chromosome^a^	Family	Individual	Total	Aligned to whole genome (%*)	Aligned to candidate regions (%**)	Coverage in %	Mean	Median
**3**	**27**	**07S722**	3,123,937	2,956,483 (94.64)	1,186,611 (40.14)	98.04	26	25
		**07S723**	4,922,157	4,538,392 (92.20)	1,518,625 (33.46)	98.43	29	29
		**07S724**	4,183,568	3,954,837 (94.53)	1,515,614 (38.32)	97.89	28	26
		**07S725**	2,952,969	2,839,271 (96.15)	1,168,679 (41.16)	97.11	24	22
	**60**	**06-240**	2,652,926	2,580,914 (97.29)	882,837 (34.21)	97.96	22	20
		**96-652**	5,934,453	4,737,175 (79.82)	1,670,157 (35.26)	98.15	28	24
	**531**	**I-1408**	12,228,047	11,188,204 (91.50)	4,694,871 (41.96)	99.07	57	48
		**I-904**	4,293,087	3,585,982 (83.53)	1,531,322 (42.70)	97.50	30	22
	**713**	**07S635**	7,568,672	7,442,938 (98.34)	2,793,056 (37.53)	99.11	45	44
		**07S636**	7,160,552	6,889,152 (96.21)	2,574,119 (37.36)	98.94	43	42
**6**	**11**	**04-168**	5,734,052	5,599,100 (97.65)	2,459,740 (43.93)	98.57	43	42
		**96-265**	6,240,024	6,012,522 (96.35)	2,642,942 (43.96)	98.22	35	32
	**40**	**07S576**	2,006,661	1,667,648 (83.11)	779,723 (46.76)	97.11	18	17
		**07S581**	4,016,214	3,618,178 (90.09)	1,568,060 (43.34)	97.66	25	23
	**929**	**I-1627**	5,811,276	5,665,182 (97.49)	2,311,149 (40.80)	98.52	33	32
		**I-3345**	2,602,250	2,554,051 (98.15)	1,059,131 (41.47)	98.27	23	23
	**990**	**I-1927**	8,134,956	7,903,785 (97.16)	3,029,994 (38.34)	98.84	51	50
		**I-1928**	7,922,500	7,590,406 (95.81)	2,817,358 (37.12)	99.02	49	48
	**1125**	**I-2033**	2,747,911	2,666,280 (97.03)	1,105,059 (41.45)	97.87	24	23
		**I-4347**	2,517,619	2,406,505 (95.59)	1,088,350 (45.23)	97.74	24	24
		**TOTAL**	102.753.831	96,397,005 (93.81)	38,397,397 (39.83)			
		**Average Affected**	5,137,692	4,819,850 (93.63)	1,919,870 (40.22)	98.20	33	31
		**Control pool**	22,390,251	18,221,565 (81.38)	7,438,610 (40.82)	99.33	111	98
		**Average All**	5,214,336	4,775,773 (91.58)	1,909,833 (39.99)	98.25	37	34

The number of sequences and the depth values are shown for a total of 20 individuals from 9 non-BRCA1/2 families and 4 individuals from the control population (Control pool).

a chromosome in which linkage signal was found for each of the families.

*with respect to the total number of sequences, ** with respect to the numbers of sequences aligned to the whole genome.

We calculated the coverage (number of bases covered after the alignment) per individual and obtained an average of 98.25% of candidate bases covered for the affected and the control individuals (Table 1). We did not observe significant coverage differences between the candidate coding regions located on chromosomes 3 and 6. Importantly, a lack of correlation between the coverage and the total number of sequences was observed (r^2^ = 0.013, Figure S1A). However, this lack of correlation turned into a logarithmic trend when the number of sequences aligned to the candidate coding regions was used (r^2^ = 0.69, Figure S1B).

In order to know the power to confidentially detect possible causal variants, we calculated the global depth (number of sequences that cover a single base) for every base along the candidate coding regions per individual. As expected, a strong correlation (r^2^ = 0.96) was found between the global depth and the number of reads aligned to the candidate coding regions (Figure S1C). Taking into account only those bases that presented coverage (depth >0), from both affected and controls individuals, the mean and the median of the depth were 37 and 34 respectively, showing a strong correlation between them (r^2^ = 0.98, Figure S1D).

### Data quality control

#### a) Coverage homogeneity

We calculated the mean and the median of the depth for stretches of 15 bases along the candidate regions, and obtained the log-ratio of the median between each affected individual and the control pool. The mean log-ratio for the global data was −0.06 with a standard deviation of 0.55 (Table 2), showing a homogeneous distribution of the coverage between affected and control individuals. We calculated the upper and lower threshold for each individual (Table 2) and we identified several regions where the coverage differed between the affected individuals and the control pool (data not shown), most of them flanking the candidate coding regions (which are usually low coverage regions). In addition, the strong correlation (r^2^ = 0.99, Figure S1E) between the median and the mean of the coverage for these regions in the dataset supported that these differences in coverage are not due to extreme values within the same coding region but are due to chance, ruling out potential problems in the capture step.

**Table 2:** Index value parameters for coverage study.

Chr	Fam	Ind	Mean	St Dev	Upper	Lower
**3**	27	07S722	−0.13	0.75	1.12	−1.38
		07S723	−0.04	0.31	0.77	−0.86
		07S724	−0.07	0.35	0.77	−0.92
		07S725	−0.10	0.43	0.82	−1.03
	60	06-240	−0.01	0.40	0.89	−0.91
		69-652	0.00	0.59	1.10	−1.09
	531	I-1408	0.05	0.53	1.08	−0.97
		I-904	−0.31	1.83	2.02	−2.64
	713	07S635	−0.04	0.62	1.08	−1.16
		07S636	−0.04	0.45	0.92	−0.99
**6**	11	04-168	−0.09	0.35	0.76	−0.94
		96-265	−0.02	0.36	0.83	−0.88
	40	07S576	−0.05	0.73	1.18	−1.29
		07S581	0.00	0.41	0.91	−0.91
	929	I-1627	−0.02	0.36	0.83	−0.88
		I-3345	−0.09	0.36	0.77	−0.95
	990	I-1927	−0.08	0.80	1.22	−1.38
		I-1928	−0.03	0.63	1.10	−1.16
	1125	I-2033	−0.03	0.44	0.91	−0.97
		I-4347	−0.09	0.39	0.80	−0.98
		**Global**	−0.06	0.55	0.99	−1.11

In order to evaluate the quality of the coverage within the candidate coding regions, we calculated an index value (*Is*, see Material and Methods). Mean, standard deviation, and lower and upper thresholds for *Is* used in the coverage study are shown for each affected individual and for the entire set of individuals (**Global**).

#### b) Score

Genotype calling accuracy was demonstrated elsewhere (>90% of the known SNPs) by using a HapMap sample in the exon-capture report [16]. In order to test the suitability of our analysis pipeline when looking for unknown polymorphisms, we used the candidate SNPs from a single family obtained using different Depth Score (DS, see Material and Methods) thresholds for both the affected samples and the control pool data (Table 3). We observed that from DS  = 50 onwards for the samples, the DS threshold used for the control pool data had no effect on the number of candidate SNPs, highlighting the specificity of our DS. In order to be as conservative as possible, we considered that the best False Positive/False Negative (FP/FN) relationship was for a DS >50 for samples and a DS >14 for the control pool. Additionally, we performed an analysis using MAQ software in a subgroup of families and we observed that there was no correlation between MAQ variant score and Sanger confirmation (Table 4). These results demonstrated the higher accuracy of our analysis methodology compared to the algorithm used by MAQ.

**Table 3:** Depth Score threshold optimization assay.

**Chr**	6																																
**Family**	990																																
**DS_Individuals**	0			10			20			30			40			50			60			70			80			90			100		
**DS_Control Pool**	0	14	50	0	14	50	0	14	50	0	14	50	0	14	50	0	14	50	0	14	50	0	14	50	0	14	50	0	14	50	0	14	50
**Candidate SNPs before homology**	9768	15791	15880	33	60	101	12	14	27	10	10	15	9	9	12	8	8	8	7	7	7	5	5	5	3	3	3	2	2	2	1	1	1
**Candidate SNPs after homology**	–	–	–	25	43	71	7	7	16	5	5	8	4	4	6	4	4	4	4	4	4	4	4	4	3	3	3	2	2	2	1	1	1
**Confirmed by Sanger**	–	–	–	–	–	–	4	4	4	4	4	4	4	4	4	4	4	4	4	4	4	4	4	4	3	3	3	2	2	2	1	1	1
**FPR**	–	–	–	–	–	–	43	43	75	20	20	50	0	0	33	0	0	0	0	0	0	0	0	0	0	0	0	0	0	0	0	0	0
**FNR**	–	–	–	–	–	–	0	0	0	0	0	0	0	0	0	0	0	0	0	0	0	0	0	0	25	25	25	50	50	50	75	75	75

The filtering results for various DS scores in a sample family (Family 990) are shown below. We observed that the threshold used for the control pool did not affect the total number of variants identified when using a DS threshold of 50 or higher for the cases. Taking into account the false positive rates (FPR) and false negative rates (FNR), and in order to be as conservative as possible, we finally chose a DS threshold of 50 for cases and a DS threshold of 14 for the control pool data.

**Table 4:** Variant filtering results using MAQ software.

Chr	Family	Position (hg18)	Gen	Reference	Variant	MS^a^	Consequence	Sanger confirmation
3	27	170968060	TERC [MIM:602322]	G	C	255/39	NON_SYNONYMOUS_CODING	No
	531	162443032	NMD3 [MIM:611021]	A	G	16/55	NON_SYNONYMOUS_CODING	No
6	531	150094561	NUP43 [MIM:608141]	C	T	38/11	STOP_GAINED	No^b^
	713	149763387	SUMO4 [MIM:608829]	A	G	50/50	NON_SYNONYMOUS_CODING	No
	990	146761618	GRM1 [MIM:604473]	C	T	93/80	NON_SYNONYMOUS_CODING	Yes
		150205485	LRP11^c^	T	C	55/77	NON_SYNONYMOUS_CODING	Yes
		151202809	PLEKHG1^c^	G	A	63/46	NON_SYNONYMOUS_CODING	Yes^d^

A lack of correlation between MAQ score (MS) and Sanger sequencing confirmation was observed, since variants showing a MS  = 255 (maximum) were not confirmed whereas others with a MS  = 55 were validated.

a MAQ score for individual 1/individual 2.

b variant selected in a non-candidate chromosome for this family because of the truncating effect.

c No MIM reference [25].

d recently described in the Ensembl database.

### Variant identification

**Figure 1:**
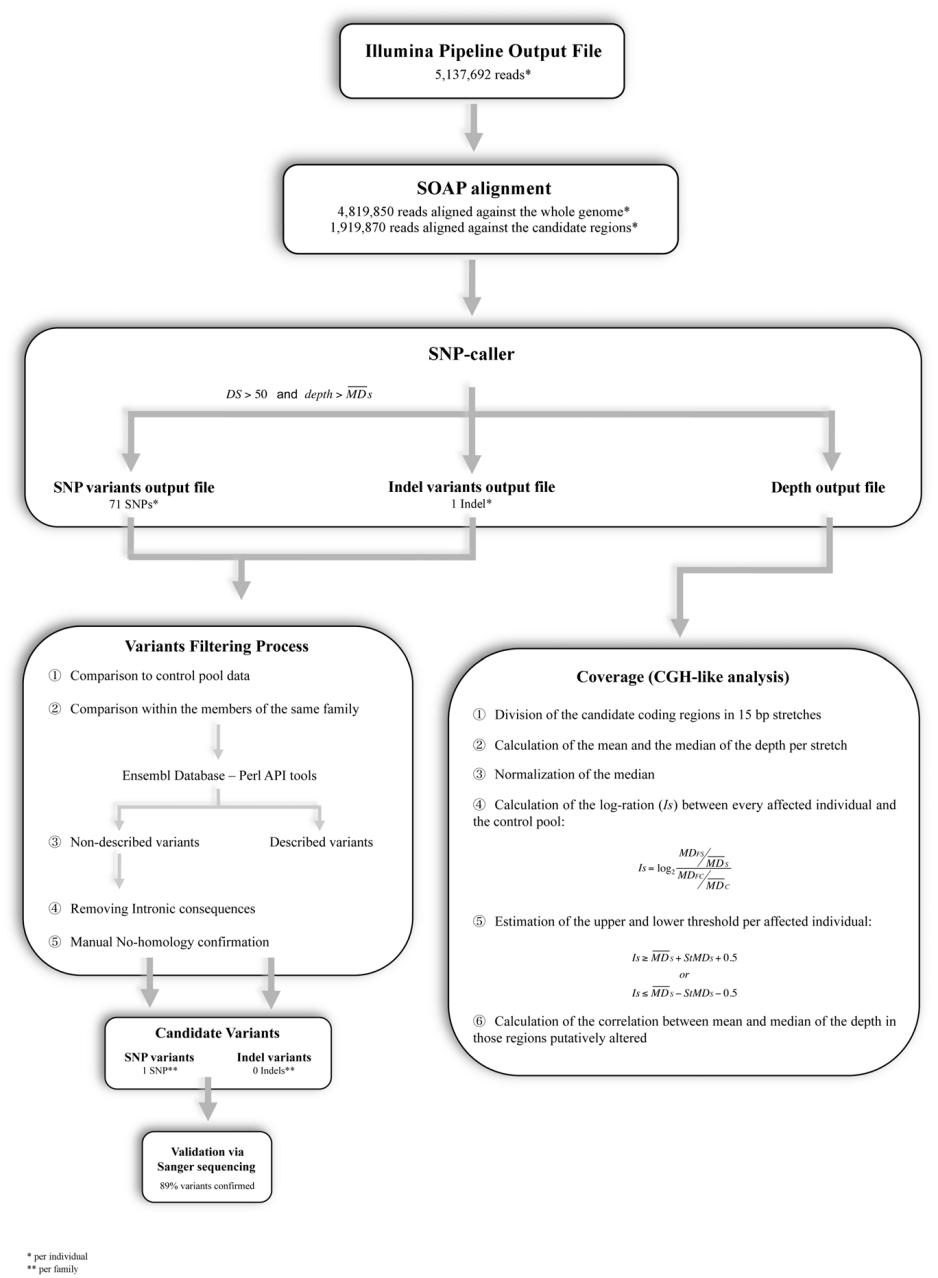
Filtering process. Analysis pipeline used in the identification of the candidate variants. Left boxes correspond to processing of variants; right box corresponds to coverage analysis. See text for details.

We included a first filter (see Material and Methods) in our SOAP-based SNP-caller to discard the maximum amount of false positives, obtaining 99% of SNP variants discarded (Figure 1). We selected only those variants located on the chromosome of interest for each individual, resulting in an average of 71 SNPs per individual (Table 5). Subsequent filters (discarding homozygous variants, comparing to controls and comparing within members of the same family) discarded almost 80% of the remaining SNPs. Then, we used a newly developed Perl script to differentiate between described and previously undescribed SNPs and also to obtain the functional consequences for each undescribed SNP, resulting in 5 undescribed variants per family on average. Since we observed that one variant could have consequences for more than one transcript/gene, subsequent filtering was performed using the consequences of the variants instead of the variants themselves. Next, we discarded intronic consequences and continued the analysis with exonic consequences only (an average of 23% of the original number of consequences). After that, each of the remaining SNPs showed only one consequence, and in the following step we checked if the remaining SNPs could be due to homology between different regions. Finally, an average of 1 (6.25%) of the SNPs shared by all the affected members of a family could be considered a strong candidate (Table 5). Finally, 8 out of 9 candidate SNPs were confirmed by Sanger sequencing, supporting a high real positive confirmation rate (0.89). Information data about variant position, gene, type of change, Alamuth prediction, and gene function from the 8 final candidate SNPs is shown in Table 6. The mean DS for confirmed variants was 115 (74–185), whereas the mean DS for ruled out variants was 56.5 (56–57), which means that our DS score is a suitable variable for the filtering process.

**Table 5:** Summary of the variant filtering process.

Chr	Family	Individual	SNPs	After control	Shared by family	Undescribed	Consequences	Exonic	Candidate SNPs (%)*
3	27	07S722	49	10	0	0	0	0	0 (0.00)
		07S723	39	2					
		07S724	45	18					
		07S725	25	4					
	60	06-240	47	18	15	7	6	3	1 (6.67)
		96-652	61	26					
	531	I-1408	38	15	5	2	1	1	0 (0.00)
		I-904	66	42					
	713	07S635	50	32	8	4	5	2	1 (12.50)
		07S636	46	24					
6	11	96_265	96	36	17	5	12	1	0 (0.00)
		04_168	81	35					
	40	07S581	93	40	26	8	32	7	3 (11.54)
		07S576	96	53					
	929	I-3345	81	28	13	3	10	3	0 (0.00)
		I-1627	75	34					
	990	I-1927	131	63	52	14	40	8	4 (7.69)
		I-1928	119	54					
	1125	I-4347	99	33	11	2	7	0	0 (0.00)
		I-2033	74	26					
		**Average**	71	30	16	5	13	3	1 (6.25)

The number of variants after each of the filtering steps is shown for the 9 non-BRCA1/2 families. The original SNPs were matched against the control pool as well as with the other member/s of the family. Previously undescribed variants were then selected and consequences obtained using PerlAPI tools. Intronic consequences were discarded and finally the variants were checked for homology.

*with respect to SNPs shared by family.

**Table 6:** Final candidate SNPs.

Chr	Family	Position (hg18)	Gene	Reference	Variant	QS^a^	DS^b^	Consequence^c^		Alamuth prediction^d^	Gene function
3	60	161301596	AC026118.17^e^	A	T	91/91	56/57	NCG			Pseudogene
	**713**	**170284589**	**EVI1** [MIM:165215]	**A**	**G**	**95/94**	**128/100**	**3UTR**			**Hematopoietic proliferation protein, related to acute myeloid leukemia**
6	**40**	**152502855**	**SYNE1** [MIM:608441]	**C**	**T**	**105/98**	**90/92**	**NSYN**		**TOL**	**A spectrin repeat containing protein expressed in skeletal and smooth muscle, and in peripheral blood lymphocytes, that localizes to the nuclear membrane**
		**151203125**	**PLEKHG1**	**C**	**T**	**103/98**	**133/146**	**SYN**	**S1186S**		**Unknown**
		**151713613**	**AKAP12** [MIM:604698]	**C**	**T**	**98/96**	**185/183**	**SYN**	**P700P**		**Scaffold protein in signal transduction, is a cell growth-related protein**
	**990**	**146761618**	**GRM1**	**C**	**T**	**101/96**	**101/81**	**NSYN**	**R584C**	**AFF**	**Metabotropic glutamate receptor**
		**150087915**	**NUP43**	**T**	**C**	**95/93**	**101/97**	**3UTR**			**Part of a nuclear pore complex, mediating bidirectional transport of macromolecules between cytoplasm and nucleus**
		**150205485**	**LRP11**	**T**	**C**	**101/101**	**74/95**	**NSYN**	**I312V**	**NDB**	**Unknown**
		**151564223**	**AL451072.14^e^**	**G**	**A**	**93/98**	**119/116**	**NCG**			**Non-coding RNA**

Variants confirmed by direct sequencing are marked in **bold**.

a) Quality Score Ind1/Ind2, b) Depth Score Ind1/Ind2, c) NCG: Non-coding gene, SYN: Synonymous, 3UTR: 3′ UTR, NSYN: Non-synonymous.

d) NDB: Not in database, AFF: predicted to affect the protein, TOL: predicted to be tolerated.

e) No MIM reference.

Regarding indel variants, we followed the same analysis pipeline as with SNP variants. Even though 12 bp gaps were allowed (maximum length allowed for an indel variant), an average of only 1.15 indels per individual were identified, and this number decreased to 0.50 after comparison to the control pool of data. Moreover, no indel was found to segregate in our family dataset. In order to rule out the possibility that a putative causal indel could be found in one member of a family but not in the other members, we checked the consequences for the remaining indels after the comparison to the control pool data for all 20 individuals from the 9 non-BRCA1/2 families. We observed a total of 8 undescribed indel variants for the 20 individuals. Three of them were located in intronic regions, two were located in 3' UTR regions, and the remaining three indels were homozygous with a global depth  = 1. Thus, no putative truncating indel was found in our individual dataset (Table S1).

## Discussion

### High-throughput sequencing

The classical candidate-gene approach turned out to be a low-efficiency tool with regard to cost and time when used for the identification of causal genes in genetic diseases, especially when there is a large number of candidate genes (dozens to hundreds). For that reason, we decided to explore the new possibilities that massive parallel sequencing brings for the analysis of hundred of genes in the same reaction. Moreover, in the present study we developed a solid analysis pipeline based on SOAP aligner for variant detection in families with a common genetic disease via high throughput sequencing data. Hybrid selection on tiling microarrays [16] was used for the enrichment of exonic sequences within two candidate regions for carrying a breast cancer susceptibility gene. We analysed the data from 20 affected individuals from 9 different non-BRCA1/2 families to perform a mutational screening of 128 known genes and, through an exhaustive filtering process, we obtained 8 variants that are currently under different genetic and functional studies (Table 6). From a technical point of view, we obtained an average of 5.14 million reads per individual, which allowed reaching a mean global depth of 33×. We observed that multiplexing of the samples by using 5-base barcodes did not affect the capture step or the sequencing process, and that it thus represents a valuable tool when the number of sequences required to confidently cover the target region is proportionally lower than the number of sequences obtained per lane.

We performed a CGH-like analysis obtaining the log-ratio between the normalised depth data from every affected individual and the control pool to confirm a homogenous distribution of the coverage along the candidate regions. Although the results showed that the distribution was very homogeneous, some differences in coverage were observed mainly in regions flanking the coding candidate regions (low coverage regions), where small and random differences in depth value may produce bigger differences in the index value. These data supported that the capture and sequencing of the candidate regions were successfully fulfilled in the sample set.

### Mutational screening

In order to identify de novo mutations, we consider that the best option is to maintain those sequences that match equally to more than one location, even though it could be a source of alignment errors due to homology between regions. For that reason, we tested two different aligners (SOAP and Mosaik), which allow the possibility of maintaining this kind of sequences. We finally decided to use SOAP v1.0 because its output file kept all the information of the input data, while being able to detect SNPs and short (gaps >1 bp) indel variants in single-end data.

The two high throughput sequencing-based mutation-detection studies published in the literature used MAQ software for SNP-calling [11], [18]. In the first study, mutation detection was performed comparing data from tumour and skin tissues from a single leukaemia patient (AML [MIM 601626])[11]. The analysis pipeline showed a high false positive rate (87.4%) when trying to confirm final candidate SNPs. This high false positive rate suggested that more stringent conditions were necessary to filter the variants. For that reason, we designed two scores based on intrinsic variant features such as base quality (QS) and depth (DS), which were used as a first filter in our SOAP-based SNP-caller. Although more than 99% of the variants were discarded with this filter, the number of remaining variants suggested the need for further refinement. For that reason, we developed a comparison-based pipeline as previously described (see Material and Methods: Data analysis and Figure 1). Only 1 SNP (less than 1.5% of the variants detected in each individual, and around 6% of the variants shared by the same family) per family on average passed this filtering process, showing that, even after using restrictive thresholds, additional information has to be used to select the most probable variants (Table 5). Candidate SNPs were Sanger sequencing validated, and finally 8 of the 9 SNPs (89%) were confirmed. These variants had a minimum depth of 19× and a maximum depth of 155×, of which between 9 and 70 reads carried the variant. Quality scores were all close to 100, corroborating Solexa's good base-call quality in those positions. The 8 variants, which are currently under study (including functional characterization), are located in different genes (Table 6) and could be considered as the final candidate SNPs. The confirmation rate (0.89) and the lack of putatively causal mutations wrongly discarded in the filtering process (Table S2) suggested that our restrictive analysis pipeline successfully identified real previously undescribed variants.

In the second mutational screening study performed using high-throughput technology and MAQ software, the authors set up a global exome-capture method based on microarrays and a specific analysis pipeline, which was conceptually quite similar to ours, to identify causal variants for a monogenic disease [18]. The analysis pipeline was based on comparisons between data from affected individuals, HapMap individuals, and the dbSNP database, considering as candidates those functional variants that were not present in HapMap sample data nor in the dbSNP database. With a subset of our data, we performed a test using MAQ software and our analysis pipeline and observed no correlation between MAQ variant score and the confirmation rate (Table 4), evidencing a lack of accuracy in MAQ's algorithm.

Regarding indel variants, no indel fulfilled the criteria to be considered a candidate variant, neither in our dataset (Table S1) nor in the previously cited study. This could be due to the fact that indel discovery on single-end data is not as accurate as with the new paired-end technology, affecting the sensitivity of indel detection in both studies. Similarly, we cannot discard the possibility of missing the existence of large rearrangements due to the limitations of single-end data. Recently published studies are starting to demonstrate the efficiency of paired-end sequencing in the identification of genomic rearrangements [19], [20].

In another study, a complete mutational screening using Sanger sequencing was performed on 718 genes located on chromosome X in probandi from a set of 208 families diagnosed with X-linked mental retardation [21]. The authors obtained 1858 coding variants, among which 1814 (980 missense, 22 nonsense, 13 splice-site, and 799 synonymous) were SNPs, 3 were double SNPs (missense), and 41 were indels. However, only 18 SNPs (17 nonsense and 1 missense, less than 1% of the initial SNPs) and 15 indels located in 26 different genes resulted to be strong candidates for being involved in X-linked mental retardation. Similarly, our results showed that around 1% of the initial SNP variants obtained via high throughput sequencing could be considered candidates (Table 6). Because no truncating mutations passed our filters, further functional studies are required to assess whether any of the confirmed variants is ultimately a causal mutation, specifically those variants considered of interest because of their functional implications (missense and 3'UTR) and gene function.

In summary, we designed an integral analysis pipeline for mutational screening via SOAP v1.0 that resulted in a low false positive rate with a low probability of discarding real positive variants, with which we identified 8 candidate variants that are currently under functional characterization. We consider that the present strategy might be a valid second step for identifying high penetrance genes, specifically when the regions of interest show significant evidence of linkage.

## Materials and Methods

### Ethics Statement

All patients provided written informed consent for the collection of samples and subsequent analysis. We obtained ethics approval for this study from the ethics committees at all institutions/hospitals where participants were recruited [17]. The GEO [22] accesion number (GSE20406) for this study has been approved as well as GEO accession numbers for each of the samples (Table 7).

**Table 7:** GEO accession numbers of the raw data from each of the samples.

Family	Individual	Accesion Number
27	07S722	GSM511164
	07S723	GSM511165
	07S724	GSM511166
	07S725	GSM511167
60	06-240	GSM511168
	96-652	GSM511169
531	I-1408	GSM511170
	I-904	GSM511171
713	07S635	GSM511172
	07S636	GSM511173
11	96-265	GSM511175
	04-168	GSM511174
40	07S581	GSM511177
	07S576	GSM511176
929	I-3345	GSM511179
	I-1627	GSM511178
990	I-1927	GSM511180
	I-1928	GSM511181
1125	I-4347	GSM511183
	I-2033	GSM511182
Control pool		GSM511184

### Samples and candidate regions

As stated earlier, in a previous study we genotyped a total of 132 individuals from 41 non-BRCA1/2 families with almost 6,000 SNP markers, and we observed a linkage profile showing several candidate regions. Suggestive linkage signals (HLOD >2.2) were found in two regions located on chromosomes 3 and 6, which span 10.8 and 6.5 Mb, respectively, and we found 6 and 5 families putatively linked to each region [17].

In the present study, 10 of these 11 families were selected based on the availability of DNA (from at least 2 affected members per family for a total of 22 DNA samples collected) to perform a mutational screening via massive parallel sequencing. One family (Family 21) was excluded from the final analysis because the DNA library preparation of one of the members failed. However, putatively truncating mutations (e.g. new stop codons or modifications within essential splice sites, and indel variants) were analysed for the remaining individual of this family (see Table S1).

We also sequenced DNA of 4 healthy individuals from Spanish control population, which were pooled into a single control data file. This pooled-control design presented several advantages, namely reduced repercussion of differential sample DNA degradation, increased sample heterozygosity, and a balancing effect over the number of reads per sample in the final control data, leading to increased data homogeneity.

### Sequencing and exon-capture

We used the Illumina single-end technique to perform the sequencing process and an exon-capture protocol recently published [16], which was divided into two steps:

#### a) Library construction

Genomic DNA (2.5 µg) was fragmented by a triple sonication step. To optimise the sequencing process, we used 5-base barcodes (#1: 5′-GATCT-3′; #2: 5′-ATCGT-3′; #3: 5′-TGTCT3′; #4: 5′-GTGAT-3′) linked to the ligated oligos, in order to be able to pool 3 or 4 individuals per array and to discriminate the sequences obtained from each individual. After ligating the modified adaptors, 150–300 bp fragments were selected by agarose gel electrophoresis and purified. To obtain a suitable amount of product, multiple parallel PCR reactions were carried out per sample.

#### b) Exon capture

A total of 142,983 60-mer probes were designed to cover the 787 exonic regions and immobilized on high-density tiling arrays, in order to capture the coding sequences of the 128 genes located in both candidate regions. We used 14 arrays to perform the enrichment step for all the samples. A final amount of 20 µg of PCR product from 3–4 individuals was hybridised onto the array, with several blocking agents, during 65 hours (see Figure 1). After amplification of the eluted DNA, the enrichment was validated by quantitative PCR (qPCR) using the product from 4 different arrays.

### Data analysis

#### a) Alignment

After the identification of the sequences belonging to each individual and before the alignment, we removed the first five bases of every sequence corresponding to each of the barcodes. We selected SOAP v1.0 to analyse our dataset using the following parameters: a seed of 12 positions, gaps allowed up to 12 bases, a maximum of 2 mismatches, and a maximum of 5 repeated regions (reporting every region on which the sequences matched equally), using the whole genome as reference sequence. One of the main advantages of SOAP v1.0 is its ability to identify short insertions and deletions (indels) in single-end data, while maintaining all input information in the output file. We developed a specific SNP caller written in the Perl programming language to extract the information contained in the SOAP output file. The whole analysis pipeline for re-sequencing data is shown in Figure 1.

#### b) Coverage

To evaluate the homogeneity of the coverage, we calculated the mean and the median of the depth for stretches of 15 bases along the candidate regions. For each sample, we obtained the index value (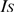) for each 15-base fragment as:
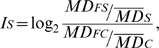



where 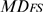 and 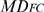 are the median of the depth in a 15-base fragment for the sample and the control pool respectively, and 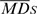 and 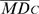 are the global median of the depth for the sample and the control pool respectively. To select supposedly altered regions, we calculated the upper and the lower thresholds as:
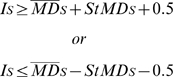



where 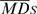 is the global median and 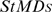 the standard deviation of the depth for the sample. Finally, for these putatively altered regions, we calculated the correlation between the mean and the median of the depth to evaluate possible coverage gaps within them.

#### c) Scores

To further select candidate heterozygous variants, we calculated the mean quality (base-calling quality from Illumina Genome Analyser) and the allele depth (number of different sequences in which an allele appeared) for both the variant and the reference alleles, and also the global depth (number of different sequences that cover a single base). We calculated two different scores based on the same mathematical formula: 
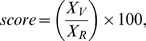



where *X_V_* represents the mean quality (for Quality Score calculations) or the allele depth (for Depth Score calculations) of the variant allele, and *X_R_* represents in each case the same parameter but for the reference allele. Scores close to 100 indicate that both alleles are equally represented.

In order to determine the optimal DS threshold for the analysis, we performed a study using the information from a single family (Table 3). We used different DS score values for the affected individuals of Family 990 and for the control pool data. We observed that the number of final candidate variants depended mostly on the threshold used for the sample data, although the false positive rate increased when using a DS threshold of 50 for the control data. Taking into account the optimal False Positive/False Negative detection rate and being as conservative as possible, we finally selected a DS threshold of 50 for the samples and a DS threshold of 14 for the control pool data (Table 3).

#### d) Variants

In the pipeline analysis (Figure 1), firstly those variants with a global depth < 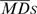 and a DS <50 were removed from the sample files as an integral function of the newly-developed SNP-caller. Homozygous variants were also discarded since we expected low-frequency heterozygous variants to be the causal variants. Similarly, variants presenting a DS <14 in the control file were discarded (see above for explanation). Only non-common variants from the previous step were selected for subsequent analysis. The next step was an intrafamilial comparison, with which variants putatively segregating in each family were obtained. Although the filtering process was performed for both SNP and indel variants, from this point onwards subsequent filters were applied to SNP variants only because no indel variants were found that fulfilled the previous conditions (Table S1). We developed a Perl tool to distinguish between described and previously undescribed variants, and also to obtain the consequences of every undescribed variant on the known transcripts, via the *Ensembl* database through the PerlAPI tools [23]. From this point onwards, intronic consequences where filtered out for each affected individual. In order to rule out possible false positives due to homology artefacts, each variant was manually checked for homology using BLAT search [24]. The following step was the confirmation of those variants that passed all filters previously mentioned via Sanger sequencing. As a final step, we used Alamut version 1.5 software to evaluate, *in silico*, how non-synonymous variants would affect the functionality of the respective candidate proteins (Figure 1).

In order to rule out the possibility that a truncating mutation was detected in one member of a family but not in the others, we analysed the truncating consequences (e.g. stop-gains and alterations in essential splice sites) of the remaining SNPs after comparison against the control pool data (Table S2).

Finally, we compared the sensitivity of our scores with a published reference, and for that we followed our analysis pipeline using MAQ software in a subset of families applying no threshold for MAQ variant score (Table 4).

## Supporting Information

Figure S1 Correlations. Coverage along the candidate regions was very high (98% on average) and no correlation between coverage and the number of sequences obtained per individual was observed (A), although we observed a logarithmic trend when the number of sequences aligned to the candidate regions was used (B). On the other hand, a strong correlation between the number of sequences aligned to the candidate coding regions and the mean depth was observed in our dataset (C). Failures in the capture step were discarded since high correlations between the global mean and the global median of the depth per individual (D) and between the mean and the median of the depth in putatively altered 15-bp regions for all the individuals (E) were observed (see text for details).
(0.13 MB DOCX)

Table S1 Indel variant filtering process.
(0.05 MB DOCX)

Table S2 Putative truncating variants discarded during the filtering process. A. Summary of the whole list of truncating variants (STOP_GAINED and ESSENTIAL_SPLICE_SITE) in some individuals after comparison to the control pool data. The original list has been filtered (Depth Score > 50) for simplicity, given that variants showing a Depth Score below the threshold are likely to be false positives. As evidenced in the table, none of the variants above the threshold has a high Global Depth value, which paired to the fair Depth Score means that in every case the variant allele was detected in a low proportion in relation to the respective reference allele. Additionally, this table shows that no putative candidate truncating variants were discarded during the filtering process, reassuring that filtering using Depth Score and Global Depth is a stringent but adequate filtering step. In conclusion, the low Global Depth and Depth Scores explain why these variants are likely to be false positive results and they were therefore excluded from the final candidate SNP list (Table 6). B. The list of truncating variants for sample 05_980. The other member of this family failed in the library preparation step, but we still performed the analysis of the variants. The most likely variant (in red) was ruled out through Sanger sequencing.
(0.15 MB DOCX)
