# Supplementary material for: Deep Sequencing of Target Linkage Assay-Identified Regions in Familial Breast Cancer: Methods, Analysis Pipeline and Troubleshooting
Source: PLoS One. 2010 Apr 2;5(4):e9976. doi: 10.1371/journal.pone.0009976 (PMC2848842; doi:10.1371/journal.pone.0009976)

**Figure S1: Correlations.**

Coverage along the candidate regions was very high (98% on average) and no correlation between coverage and the number of sequences obtained per individual was observed (A), although we observed a logarithmic trend when the number of sequences aligned to the candidate regions was used (B). On the other hand, a strong correlation between the number of sequences aligned to the candidate coding regions and the mean depth was observed in our dataset (C). Failures in the capture step were discarded since high correlations between the global mean and the global median of the depth per individual (D) and between the mean and the median of the depth in putatively altered 15-bp regions for all the individuals (E) were observed (see text for details).


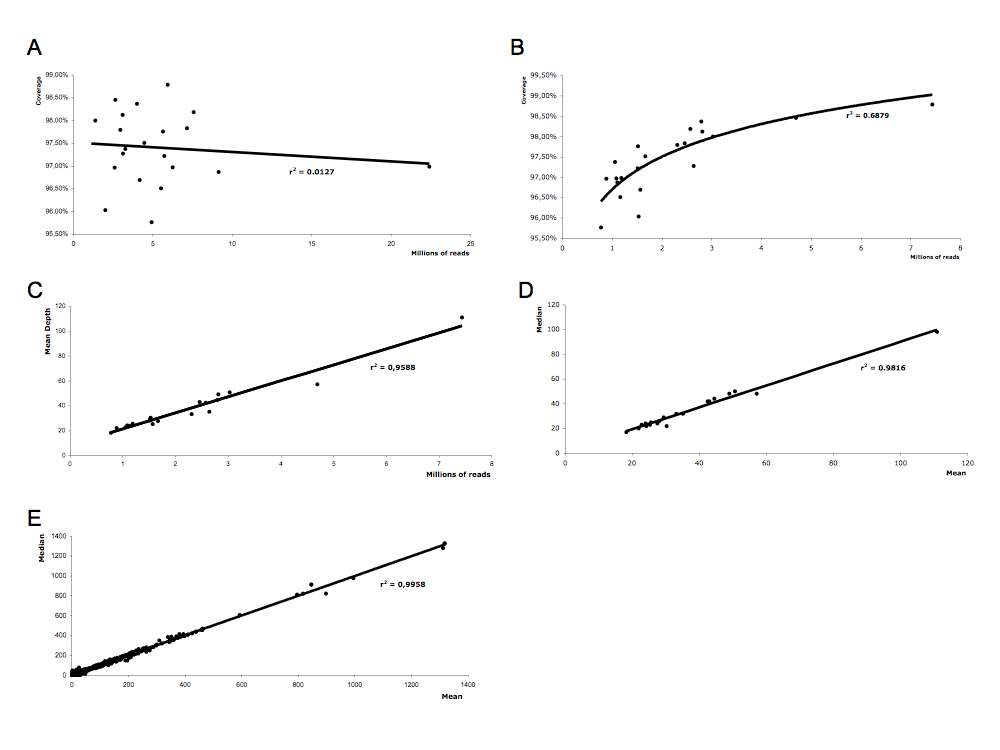

Supplement: Figure S1 — Correlations. Coverage along the candidate regions was very high (98% on average) and no correlation between coverage and the number of sequences obtained per individual was observed (A), although we observed a logarithmic trend when the number of sequences aligned to the candidate regions was used (B). On the other hand, a strong correlation between the number of sequences aligned to the candidate coding regions and the mean depth was observed in our dataset (C). Failures in the capture step were discarded since high correlations between the global mean and the global median of the depth per individual (D) and between the mean and the median of the depth in putatively altered 15-bp regions for all the individuals (E) were observed (see text for details). (0.13 MB DOCX) [file pone.0009976.s001.docx]
