# Supplementary material for: Deep Sequencing of Target Linkage Assay-Identified Regions in Familial Breast Cancer: Methods, Analysis Pipeline and Troubleshooting
Source: PLoS One. 2010 Apr 2;5(4):e9976. doi: 10.1371/journal.pone.0009976 (PMC2848842; doi:10.1371/journal.pone.0009976)
Supplement: Table S1 — Indel variant filtering process. (0.05 MB DOCX) [file pone.0009976.s002.docx]

**Table S1: Indel variant filtering process.**

| **Chr** | **Family** | **Individual** | **Indels** | **After control** | **Shared by family** |
| --- | --- | --- | --- | --- | --- |
| **3** | 27 | 07S722 | 1 | 0 | 0 |
|  |  | 07S723 | 2 | 0 |  |
|  |  | 07S724 | 2 | 1 |  |
|  |  | 07S725 | 1 | 1 |  |
|  | 60 | 06-240 | 0 | 0 | 0 |
|  |  | 96-652 | 1 | 0 |  |
|  | 531 | I-1408 | 1 | 0 | 0 |
|  |  | I-904 | 0 | 0 |  |
|  | 713 | 07S635 | 1 | 0 | 0 |
|  |  | 07S636 | 1 | 0 |  |
| **6** | 11 | 96_265 | 0 | 0 | 0 |
|  |  | 04_168 | 2 | 2 |  |
|  | 40 | 07S581 | 2 | 1 | 0 |
|  |  | 07S576 | 2 | 2 |  |
|  | 929 | I_3345 | 1 | 1 | 0 |
|  |  | I_1627 | 1 | 0 |  |
|  | 990 | I_1927 | 2 | 1 | 0 |
|  |  | I_1928 | 2 | 1 |  |
|  | 1125 | I_4347 | 0 | 0 | 0 |
|  |  | I_2033 | 1 | 0 |  |
|  |  |  |  |  |  |
|  |  | **Average** | 1,15 | 0,50 | 0,00 |
