# Supplementary material for: Deep Sequencing of Target Linkage Assay-Identified Regions in Familial Breast Cancer: Methods, Analysis Pipeline and Troubleshooting
Source: PLoS One. 2010 Apr 2;5(4):e9976. doi: 10.1371/journal.pone.0009976 (PMC2848842; doi:10.1371/journal.pone.0009976)
Supplement: Table S2 — Putative truncating variants discarded during the filtering process. A. Summary of the whole list of truncating variants (STOP_GAINED and ESSENTIAL_SPLICE_SITE) in some individuals after comparison to the control pool data. The original list has been filtered (Depth Score > 50) for simplicity, given that variants showing a Depth Score below the threshold are likely to be false positives. As evidenced in the table, none of the variants above the threshold has a high Global Depth value, which paired to the fair Depth Score means that in every case the variant allele was detected in a low proportion in relation to the respective reference allele. Additionally, this table shows that no putative candidate truncating variants were discarded during the filtering process, reassuring that filtering using Depth Score and Global Depth is a stringent but adequate filtering step. In conclusion, the low Global Depth and Depth Scores explain why these variants are likely to be false positive results and they were therefore excluded from the final candidate SNP list (Table 6). B. The list of truncating variants for sample 05_980. The other member of this family failed in the library preparation step, but we still performed the analysis of the variants. The most likely variant (in red) was ruled out through Sanger sequencing. (0.15 MB DOCX) [file pone.0009976.s003.docx]

**Table S2: Putative truncating variants discarded during the filtering process.**

A. Summary of the whole list of truncating variants (STOP_GAINED and essential_splice_site) in some individuals after comparison to the control pool data. The original list has been filtered (Depth Score > 50) for simplicity, given that variants showing a Depth Score below the threshold are likely to be false positives. As evidenced in the table, none of the variants above the threshold has a high Global Depth value, which paired to the fair Depth Score means that in every case the variant allele was detected in a low proportion in relation to the respective reference allele. Additionally, this table shows that no putative candidate truncating variants were discarded during the filtering process, reassuring that filtering using Depth Score and Global Depth is a stringent but adequate filtering step. In conclusion, the low Global Depth and Depth Scores explain why these variants are likely to be false positive results and they were therefore excluded from the final candidate SNP list (Table 6).

B. The list of truncating variants for sample 05_980. The other member of this family failed in the library preparation step, but we still performed the analysis of the variants. The most likely variant (in red) was ruled out through Sanger sequencing.

**A**

| **Family** | **Individual** | **Chromosome** | **Position** | **Gen** | **Reference allele** | **Variant allele** | **Genotype** | **QS** | **DS** | **Consequence** | **Global Depth** |
| --- | --- | --- | --- | --- | --- | --- | --- | --- | --- | --- | --- |
| 60 | 06_240 | 3 | 162442989 | NMD3 | G | T | G/T | 80 | 50 | ESSENTIAL_SPLICE_SITE | 9 |
|  | 06_240 | 3 | 166262966 | SI | C | A | C/A | 73 | 50 | ESSENTIAL_SPLICE_SITE | 3 |
|  | 06_240 | 3 | 168534468 | ZBBX | C | A | C/A | 81 | 50 | ESSENTIAL_SPLICE_SITE | 3 |
|  | 06_240 | 6 | 146281062 | SHPRH | C | A | C/A | 88 | 100 | ESSENTIAL_SPLICE_SITE | 2 |
|  | 06_240 | 6 | 147039141 | C6orf103 | G | T | G/T | 77 | 57 | ESSENTIAL_SPLICE_SITE | 11 |
|  | 06_240 | 6 | 147625118 | STXBP5 | G | T | G/T | 79 | 66 | ESSENTIAL_SPLICE_SITE | 10 |
|  | 06_240 | 6 | 147872067 | SAMD5 | C | T | C/T | 77 | 50 | STOP_GAINED | 3 |
|  | 06_240 | 6 | 151228808 | MTHFD1L | C | T | C/T | 105 | 50 | STOP_GAINED | 3 |
| 531 | I_904 | 3 | 171238698 | GPR160 | G | T | G/T | 110 | 50 | ESSENTIAL_SPLICE_SITE | 3 |
|  | I_904 | 6 | 148706054 | SASH1 | T | C | T/C | 59 | 100 | ESSENTIAL_SPLICE_SITE | 2 |
|  | I_904 | 6 | 151857015 | C6orf97 | T | G | T/G | 76 | 50 | ESSENTIAL_SPLICE_SITE | 3 |
|  | I_904 | 3 | 161426731 | AC112641.3 | C | A | C/A | 117 | 100 | STOP_GAINED | 2 |
| 27 | 07S722 | 3 | 161601302 | SMC4 | A | C | A/C | 107 | 50 | ESSENTIAL_SPLICE_SITE | 3 |
|  | 07S722 | 3 | 161601303 | SMC4 | G | C | G/C | 92 | 50 | ESSENTIAL_SPLICE_SITE | 3 |
|  | 07S722 | 3 | 162305379 | B3GALNT1 | T | G | T/G | 54 | 50 | ESSENTIAL_SPLICE_SITE | 6 |
|  | 07S723 | 3 | 162303593 | B3GALNT1 | A | G | A/G | 56 | 100 | ESSENTIAL_SPLICE_SITE | 2 |
|  | 07S723 | 3 | 166263999 | SI | G | A | G/A | 102 | 50 | STOP_GAINED | 3 |
|  | 07S725 | 3 | 161483097 | IFT80 | T | A | T/A | 65 | 50 | ESSENTIAL_SPLICE_SITE | 3 |
|  | 07S725 | 3 | 166197219 | SI | C | A | C/A | 72 | 50 | ESSENTIAL_SPLICE_SITE | 3 |
|  | 07S725 | 3 | 171460450 | PRKCI | G | T | G/T | 89 | 50 | ESSENTIAL_SPLICE_SITE | 6 |
|  | 07S725 | 6 | 146177430 | FBXO30 | A | T | A/T | 59 | 50 | ESSENTIAL_SPLICE_SITE | 3 |
|  | 07S725 | 6 | 146308467 | SHPRH | C | A | C/A | 75 | 53 | ESSENTIAL_SPLICE_SITE | 23 |
|  | 07S725 | 6 | 150001390 | KATNA1 | C | A | C/A | 80 | 57 | ESSENTIAL_SPLICE_SITE | 11 |
|  | 07S725 | 3 | 168566393 | ZBBX | G | T | G/T | 81 | 133 | STOP_GAINED | 7 |
|  | 07S725 | 3 | 169247461 | GOLIM4 | C | A | C/A | 78 | 50 | STOP_GAINED | 3 |
|  | 07S725 | 6 | 147007611 | C6orf103 | G | T | G/T | 74 | 50 | STOP_GAINED | 3 |
| 11 | 96_265 | 3 | 161477961 | IFT80 | C | A | C/A | 57 | 50 | ESSENTIAL_SPLICE_SITE | 3 |
| 40 | 07S576 | 3 | 166247495 | SI | C | A | C/A | 71 | 100 | ESSENTIAL_SPLICE_SITE | 8 |
|  | 07S576 | 3 | 168534468 | ZBBX | C | A | C/A | 129 | 50 | ESSENTIAL_SPLICE_SITE | 3 |
|  | 07S576 | 6 | 149680846 | MAP3K7IP2 | T | G | T/G | 66 | 50 | ESSENTIAL_SPLICE_SITE | 3 |
|  | 07S576 | 6 | 151228880 | MTHFD1L | T | G | T/G | 107 | 100 | ESSENTIAL_SPLICE_SITE | 2 |
|  | 07S576 | 3 | 168728494 | WDR49 | G | T | G/T | 56 | 50 | STOP_GAINED | 3 |
|  | 07S581 | 6 | 150251497 | RAET1E | C | A | C/A | 108 | 100 | ESSENTIAL_SPLICE_SITE | 2 |
|  | 07S581 | 3 | 168560381 | ZBBX | C | A | C/A | 130 | 100 | STOP_GAINED | 2 |
|  | 07S581 | 6 | 150506161 | PPP1R14C | C | A | C/A | 68 | 50 | STOP_GAINED | 3 |
| 990 | I_1927 | 6 | 149680846 | MAP3K7IP2 | T | G | T/G | 85 | 50 | ESSENTIAL_SPLICE_SITE | 3 |
|  | I_1927 | 6 | 151754247 | ZBTB2 | C | A | C/A | 88 | 50 | ESSENTIAL_SPLICE_SITE | 3 |
| 1125 | I_2033 | 6 | 147625118 | STXBP5 | G | T | G/T | 68 | 66 | ESSENTIAL_SPLICE_SITE | 5 |
|  | I_2033 | 6 | 147672912 | STXBP5 | G | T | G/T | 68 | 100 | ESSENTIAL_SPLICE_SITE | 4 |
|  | I_4347 | 3 | 161736380 | KPNA4 | C | A | C/A | 69 | 50 | ESSENTIAL_SPLICE_SITE | 4 |
|  | I_4347 | 3 | 166247495 | SI | C | A | C/A | 73 | 50 | ESSENTIAL_SPLICE_SITE | 6 |
|  | I_4347 | 6 | 147677092 | STXBP5 | G | T | G/T | 59 | 100 | ESSENTIAL_SPLICE_SITE | 2 |

**B**

| **Family** | **Individual** | **Chromosome** | **Position (hg18)** | **Gen** | **Reference allele** | **Variant allele** | **Genotype** | **QS** | **DS** | **Consequence** | **Global**  **Depth** |
| --- | --- | --- | --- | --- | --- | --- | --- | --- | --- | --- | --- |
| **21** | 05_980 | 3 | 160965047 | SCHIP1 | C | A | C/A | 98 | 26 | STOP_GAINED | 34 |
|  |  | 3 | 166192869 | SI | C | A | C/A | 71 | 26 | STOP_GAINED | 19 |
|  |  | 6 | 146797238 | GRM1 | C | A | C/A | 65 | 33 | STOP_GAINED | 4 |
|  |  | 6 | 151754247 | ZBTB2 | C | A | C/A | 69 | 25 | ESSENTIAL_SPLICE_SITE | 6 |
|  |  | 6 | 151754247 | ZBTB2 | C | T | C/T | 74 | 25 | ESSENTIAL_SPLICE_SITE | 6 |
